# Infectious diseases in perspective

**DOI:** 10.1186/s12879-016-1395-4

**Published:** 2016-03-08

**Authors:** Anca Streinu-Cercel

**Affiliations:** Carol Davila University of Medicine and Pharmacy, Bucharest, Romania; National Institute for Infectious Diseases “Prof. Dr. Matei Balș”, Bucharest, Romania

Infectious diseases represent an ever-changing field of practice. Virtually each week brings new information, some with the potential to adjust or reinvent the current clinical practice. It is our task as clinicians to browse through field literature, through reports of randomized controlled trials, excerpts of conference proceedings, and to choose relevant scoops with high applicability in patient management.

This supplement brings together four articles relevant to four different areas of medical practice, from HIV infection to Gram-negative bacilli such as *Acinetobacter baumannii* and *Pseudomonas aeruginosa*, and interdisciplinary studies on infectious acute maxillary sinusitis.

Non-infectious comorbidities are of utmost importance in the management of HIV infection, as occurrence of kidney impairment, or fractures following decrease in bone mineral density, can significantly impact the quality of life and/or even life expectancy. The study presented at the 11^th^ Edition of the Scientific Days of the National Institute for Infectious Diseases “Prof. Dr. Matei Balș” describes the epidemiology of chronic kidney disease and bone demineralization in HIV-positive patients from the Romanian HIV cohort, patients who acquired HIV infection at an early age, in infancy and childhood, in the late ‘80s, and who have since have tried all available therapeutic options. The prevalence of chronic kidney diseases is reported at 29.6 % for stage 2 and 1.4 % for stage 3 chronic kidney disease. The prevalence of lumbar osteopenia and osteoporosis was 33.3 % and 13.7 %, respectively, and that of femoral osteopenia and osteoporosis was 37.3 % and 7.8 %, respectively.

An in-depth study of the virulence factors expressed by *Pseudomonas aeruginosa* revealed that virulence genes are highly prevalent in strains isolated from patients treated in a Dermatology department in Bucharest, Romania, namely *ExoT* (100 %), *AlgD* (92.3 %), genes codifying for phospholipases (84.6 % each) and genes coding for protease IV (61.5 %).

A study performed by oromaxillofacial and dentoalveolar surgeons revealed an incidence of 4.3 % acute maxillary sinusitis in patients undergoing sinus lift procedures, and identified certain grafting materials which may be correlated with higher incidences. This study can pave the way for new therapeutic guidelines for the interdisciplinary management of acute sinusitis, which is currently treated by dental surgeons, ear-nose-throat practitioners, and infectious diseases specialists, alike.

The last study included in the volume evaluated the patterns of antimicrobial susceptibility of *Acinetobacter baumannii* strains isolated from patients in intensive care, and reported a worrisome prevalence of carbapenem resistance: 94.6 % to imipenem and 86.5 % to meropenem. The authors conclude that there is dire need for new therapeutic options, as carbapenems are considered last-resort antibiotics, and the only antimicrobial to which *Acinetobacter* strains still displayed satisfactory susceptibility was colistin, with resistance reported in 9/37 strains (24.3 %) in this study.

